# SARS-CoV-2 causes senescence in human cells and exacerbates the senescence-associated secretory phenotype through TLR-3

**DOI:** 10.18632/aging.203560

**Published:** 2021-09-16

**Authors:** Utkarsh Tripathi, Rayhane Nchioua, Larissa G. P. Langhi Prata, Yi Zhu, Erin O. Wissler Gerdes, Nino Giorgadze, Tamar Pirtskhalava, Erik Parker, Ailing Xue, Jair Machado Espindola-Netto, Steffen Stenger, Paul D. Robbins, Laura J. Niedernhofer, Stephanie L. Dickinson, David B. Allison, Frank Kirchhoff, Konstantin Maria Johannes Sparrer, Tamar Tchkonia, James L. Kirkland

**Affiliations:** 1 Robert and Arlene Kogod Center on Aging, Mayo Clinic, Rochester, MN 55905, USA; 2 Institute of Molecular Virology, Ulm University Medical Center, Ulm 89081, Germany; 3 Department of Physiology and Bioengineering, Mayo Clinic, Rochester, MN 55905, USA; 4 Institute for Medical Microbiology and Hygiene, Ulm University Medical Center, Ulm 89081, Germany; 5 Institute on the Biology of Aging and Metabolism, Department of Biochemistry, Molecular Biology and Biophysics, University of Minnesota, Minneapolis, MN 55455, USA; 6 Department of Epidemiology and Biostatistics, School of Public Health, Indiana University-Bloomington, Bloomington, IN 47405, USA; 7 Division of General Internal Medicine, Department of Medicine, Mayo Clinic, Rochester, MN 55905, USA

**Keywords:** SARS-COV-2, senescence, toll like receptor 3, COVID-19

## Abstract

Senescent cells, which arise due to damage-associated signals, are apoptosis-resistant and can express a pro-inflammatory, tissue-destructive senescence-associated secretory phenotype (SASP). We recently reported that a component of the severe acute respiratory syndrome coronavirus 2 (SARS-CoV-2) surface protein, S1, can amplify the SASP of senescent cultured human cells and that a related mouse β-coronavirus, mouse hepatitis virus (MHV), increases SASP factors and senescent cell burden in infected mice. Here, we show that SARS-CoV-2 induces senescence in human non-senescent cells and exacerbates the SASP in human senescent cells through Toll-like receptor-3 (TLR-3). TLR-3, which senses viral RNA, was increased in human senescent compared to non-senescent cells. Notably, genetically or pharmacologically inhibiting TLR-3 prevented senescence induction and SASP amplification by SARS-CoV-2 or Spike pseudotyped virus. While an artificial TLR-3 agonist alone was not sufficient to induce senescence, it amplified the SASP in senescent human cells. Consistent with these findings, lung p16^INK4a+^ senescent cell burden was higher in patients who died from acute SARS-CoV-2 infection than other causes. Our results suggest that induction of cellular senescence and SASP amplification through TLR-3 contribute to SARS-CoV-2 morbidity, indicating that clinical trials of senolytics and/or SASP/TLR-3 inhibitors for alleviating acute and long-term SARS-CoV-2 sequelae are warranted.

## INTRODUCTION

The current COVID-19 pandemic has illuminated the vulnerability of the elderly and those with chronic diseases to increased SARS-CoV-2-mediated mortality. By August 2021, there had been over 207 million cases of SARS-CoV-2 and 4.3 million deaths worldwide (https://en.wikipedia.org/wiki/Template: COVID-19_pandemic_data accessed 08/16/2021). Those over age 65 accounted for 45% of patients hospitalized and 80% of those who died with SARS-CoV-2 [1]. Recent studies have estimated that between 10-30% of patients experience persistent symptoms months after resolution of acute cases of COVID-19. These prolonged symptoms are referred to as post-acute sequelae of SARS-CoV-2 infection (PASC) and might be a consequence of chronic inflammation induced during the acute phase of infection [2]. A mechanistic level of understanding of the short- and long-term effects of SARS-CoV-2 infection on cell and tissue function is urgently needed to tackle its acute and chronic adverse health outcomes.

We recently reported that senescent cells contribute to the pathogenesis of acute β-coronavirus infections [3]. The senescent cell fate entails essentially permanent cell-cycle arrest with extensive changes in cell morphology and gene expression [4–6]. Senescence in many cell types is driven by damage/danger signals as well as metabolic insults, mechanical/shear forces, hypoxia, reactive oxygen species (ROS), repeated replication, oncogenes, telomere damage, and other stressors [7–10]. Senescence is established through transcription factor cascades that can include p16^INK4a^/retinoblastoma protein and/or p53/p21^CIP1^, which induce extensive changes in gene expression and organelle function, histone modifications, epigenomic remodelling, altered protein production, and profound morphologic and metabolic shifts [11, 12]. Senescent cells are resistant to apoptosis, persistent, metabolically active, and mainly cleared by the immune system [13–15]. A majority of, but not all senescent cells can develop a senescence-associated secretory phenotype (SASP), with release of inflammatory cytokines, chemokines, proteases, pro-coagulant and pro-fibrotic factors, bioactive lipids, other reactive metabolites, non-coding nucleotides, and extracellular vesicles [16–19]. The SASP can induce secondary senescence of neighboring previously non-senescent cells and even of cells at a distance [20, 21].

Senescence and the SASP have emerged as mechanisms that appear to contribute to aging phenotypes and multiple chronic conditions and diseases, even in younger individuals (*e.g*., in those with obesity/diabetes, cardiac, lung, and kidney disorders, arthritis, cancers, osteoporosis, or neurocognitive or immunological dysfunction), and can underlie adverse effects due to certain drugs and treatments, such as chemotherapy or radiation [5, 22–32]. Many of the clinical conditions linked to cellular senescence share features with complications associated with sequelae of COVID-19. Both can be associated with cognitive dysfunction, frailty/weakness, arthritis and arthralgias, cardiac conditions, and lung dysfunction and fibrosis, among others [4, 5, 23, 24, 27, 29, 31, 33–35]. Additionally, factors such as IL-6, IL-8, and IP-10 that are SASP components appear to predict the severity of SARS-CoV-2 infection [36]. These factors may also contribute to prolonged disease, hyper-inflammation/cytokine storm, acute respiratory distress (ARDS), and multi organ failure.

Incoming viral pathogens are detected by the innate immune system through dedicated sensors, Toll-Like Receptors (TLRs), which recognize pathogen-associated molecular patterns and engage innate immune responses. Although SASP-related cytokines are a critical component of the innate immune response and aid in clearing viral infections, dysregulated release of pro-inflammatory cytokines may lead to cytokine storm, which can result in severe damage to host tissues and organs. Here, we examined innate immune responses to SAR-CoV-2 and links to cellular senescence. We found that both a Spike pseudotyped virus (pseudovirus) and the genuine SARS-CoV-2 virus can induce senescence in human cells. Furthermore, the senescent cell SASP was amplified by TLR-3-dependent signaling. Clinical trials appear be warranted to ascertain if senolytics, agents that selectively eliminate senescent cells, senomorphics, which inhibit the SASP, and/or TLR-3 inhibitors can alleviate acute or long-term sequelae of SARS-CoV-2 infection.

## RESULTS

### Increased TLR-3 expression in senescent cells

**Figure 1:**
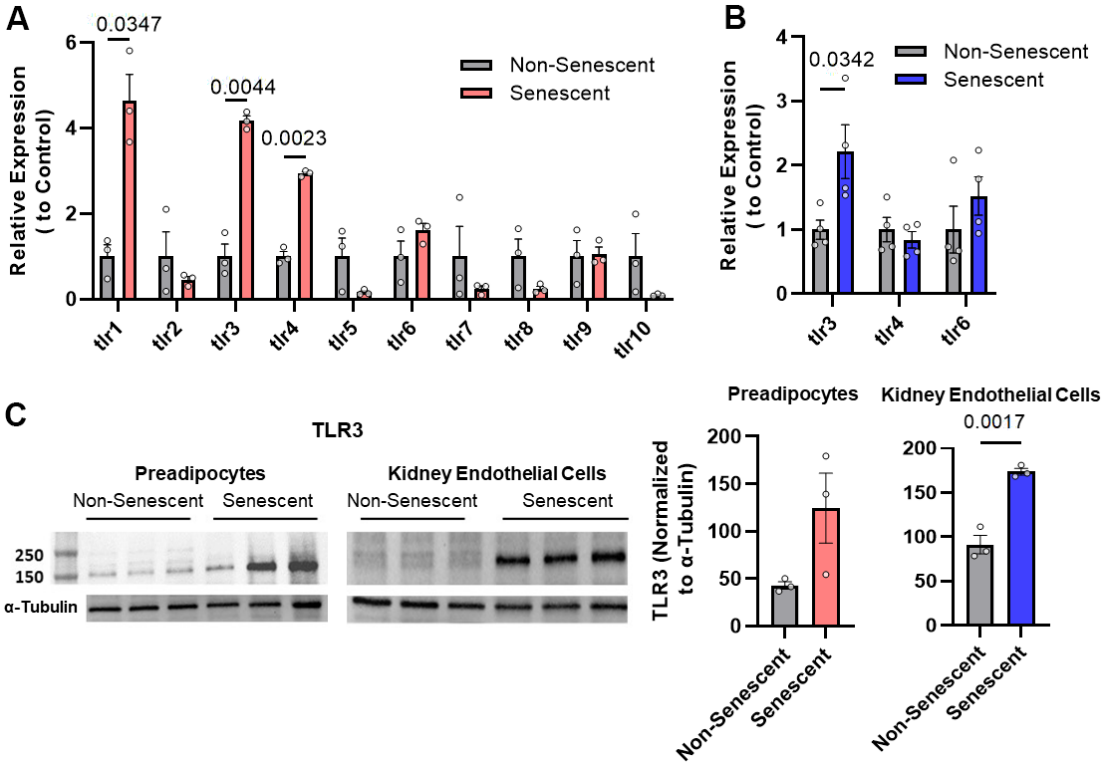
**Toll-like receptor-3 (TLR-3) is increased in senescent *vs*. non-senescent human kidney endothelial cells and preadipocytes.** (**A**) TLR expression (rtPCR) in radiation-induced senescent *vs*. non-senescent human preadipocytes (n=3). (**B**) TLR (rtPCR) in radiation-induced senescent human kidney endothelial cells (n=4) *vs*. non-senescent cells. Data are expressed as a function of non-senescent cells; mean +/- SEM, paired (**A**), unpaired (**B**), 2-tailed Student’s t-tests. (**C**) TLR-3 protein (Western blots) in non-senescent and senescent preadipocytes (n=3) and kidney endothelial cells (n=3) with tubulin as loading control (optical density of TLR-3 as a function of α-tubulin [%]). Data are expressed as a function of non-senescent cells; mean +/- SEM, paired (preadipocytes), unpaired (kidney endothelial cells), 2-tailed Student’s t-tests.

TLRs are major innate immune sensors whose expression varies depending on cell type [37]. Therefore, we analyzed if TLRs are present on senescent cells. TLR-1, -3, and -4 were more highly expressed in radiation-induced senescent *vs*. non-senescent human preadipocytes (Figure 1A). Among other ligands, TLR-1 binds gram-positive bacterial antigens, TLR-3 binds viral RNA, and TLR-4 binds lipopolysaccharide [38]. Also, TLR-3 mRNA was increased in senescent human kidney endothelial cells compared to non-senescent controls (Figure 1B). TLR-3 protein levels were higher in radiation-induced senescent human kidney endothelial cells than non-senescent controls (Figure 1C). Thus, TLR-3, a sensor of viral RNA, can be present in increased abundance on senescent cells.

### Pseudovirus induces senescence in non-senescent human kidney endothelial and lung epithelial cells

**Figure 2:**
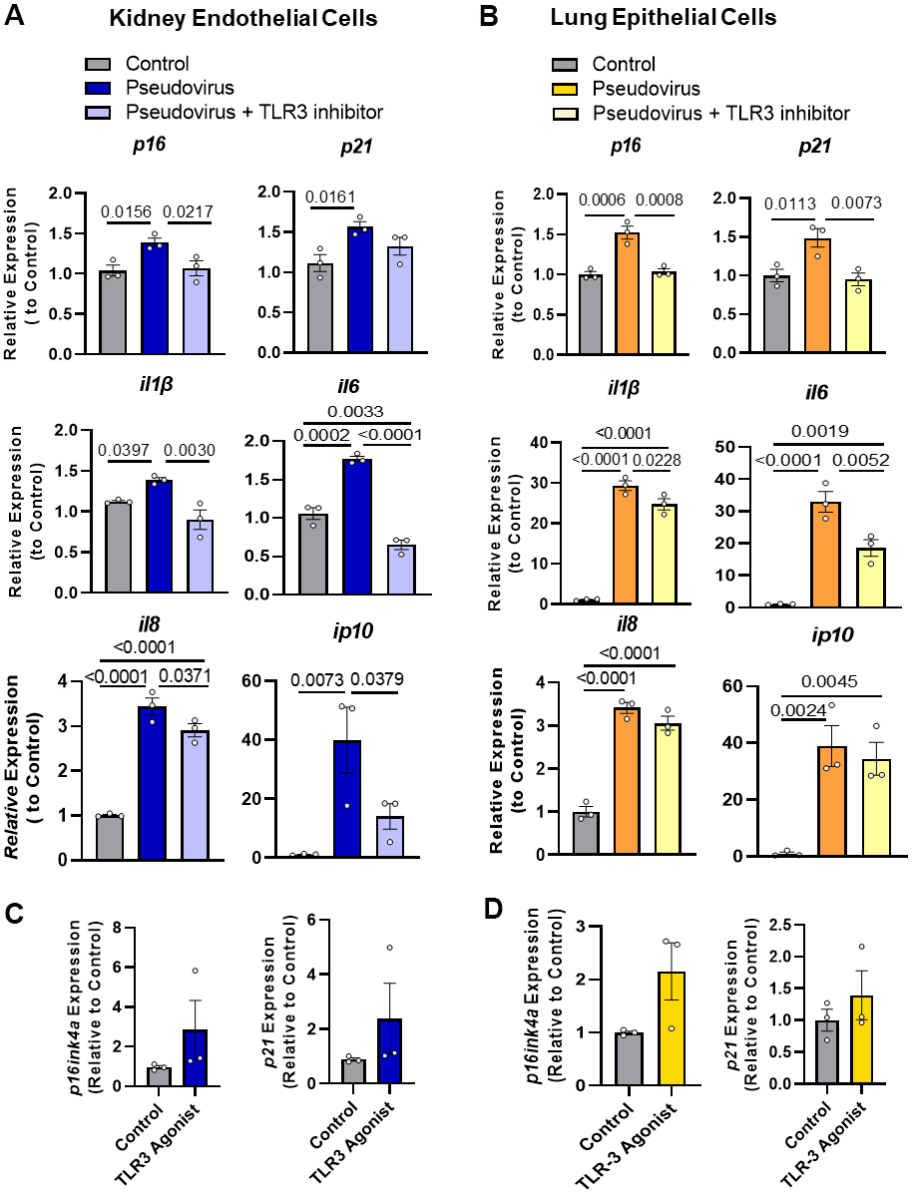
**Pseudovirus exposure increases senescence markers in non-senescent cells; these increases in markers were attenuated by TLR-3 inhibitor.** (**A**) Non-senescent kidney endothelial cells and (**B**) human lung epithelial cells had higher expression of senescence markers and SASP factors upon treatment with pseudovirus, while using TLR-3 inhibitor decreased their expression. Cells were exposed to the pseudovirus in the presence of TLR-3 inhibitor or vehicle (control) for 14 days. (**C**, **D**) Activating TLR-3 was not sufficient to induce senescence: TLR-3 agonist did not induce senescence as extensively as the pseudovirus. Non-senescent kidney endothelial (**C**) and lung epithelial cells (**D**) were treated with the TLR-3 agonist Poly I:C (2 and 10μg/ml, respectively), for 7 days or pseudovirus, when senescence markers and SASP factor expression were measured. Data are expressed as a function of untreated non-senescent cells; mean +/- SEM, 1-way ANOVA and *post hoc* comparisons with Fisher’s LSD (**A**, **B**) and unpaired Student’s t-tests (**C**, **D**).

**Figure 3:**
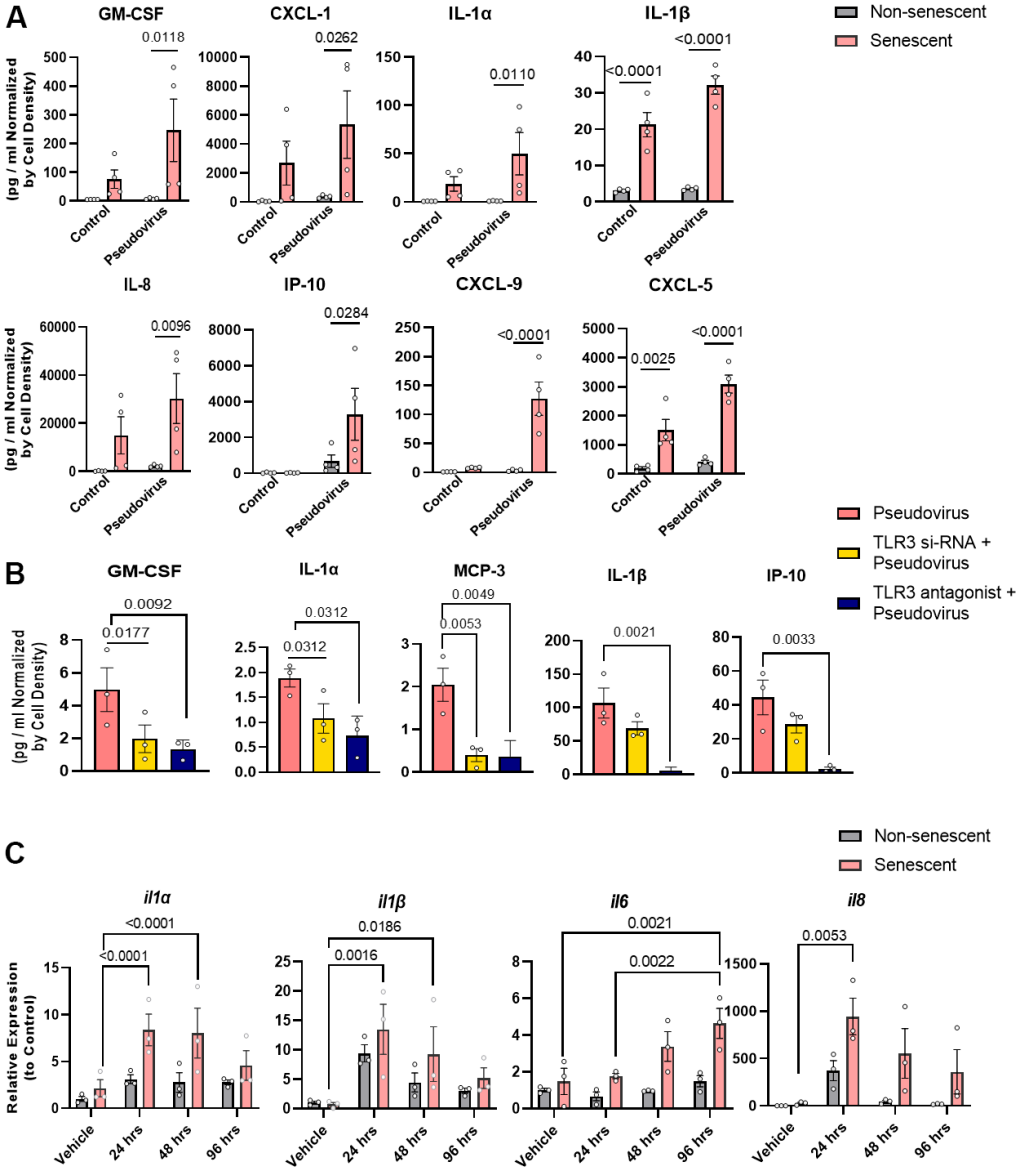
**Pseudovirus amplifies the SASP in senescent preadipocytes and genetically or pharmacologically inhibiting TLR-3 attenuates this SASP amplification.** (**A**) SASP factors were assayed in senescent and non-senescent preadipocytes (n=4) treated with pseudovirus for 96 hrs. Data are shown as a function of cell number; mean +/- SEM, 2-way repeated measures ANOVA. (**B**) Senescent cells were transfected with TLR-3 siRNA or treated with TLR-3 antagonist (10μM) and then exposed to pseudovirus for 96 hrs. Data are expressed as a function of untreated senescent cells; mean +/- SEM, repeated 1-way ANOVA and *post hoc* pairwise comparisons Fischer’s LSD. (**C**) TLR-3 agonist increases SASP factor expression in senescent (but not non-senescent) human preadipocytes. Senescent and non-senescent preadipocytes were exposed to Poly I:C for 24, 48, or 96 hrs. and analyzed for SASP factors (rtPCR). Data are expressed as a function of untreated senescent cells; mean +/- SEM, repeated 2-way ANOVA and *post hoc* pairwise comparison Tukey’s HSD. All other significant p values are listed in Supplementary Table 1.

We previously reported that mouse coronavirus induces senescence in vivo (see Figure 3B) [3]. To examine whether exposure of senescent cells to SARS-CoV-2 might induce senescence through TLR-3, non-senescent human cells were exposed to vesicular stomatitis virus (VSV) encoding SARS-CoV-2 Spike instead of its native G Glycoprotein (VSVdG*Spike), a pseudovirus that simulates the presence of SARS-CoV-2 but is safer to handle [39]. Expression of the cellular senescence markers p16^INK4a^ and p21^CIP1^ was increased by exposing non-senescent human kidney endothelial (Figure 2A) and lung epithelial cells (Figure 2B) to this pseudovirus for 14 days. The TLR-3 antagonist, (R)-2-(3-chloro-6-fluorobenzo[b]thiophene-2-carboxamido)-3-phenylpropanoic acid, counteracted this induction of senescence markers by the pseudovirus in both human kidney endothelial and lung epithelial cells (Figure 2A, 2B). Exposure to a TLR-3 agonist, polyinosine-polycytidylic acid (Poly I:C), for 1 week tended to induce increase in p16^INK4a^ and p21^CIP1^ expression in human non-senescent preadipocytes, but not significantly (Figure 2C). Taken together, these results indicate that pseudovirus sensed by TLR-3 is sufficient to promote senescence.

### Pseudovirus amplifies the SASP of senescent human cells *via* TLR-3

Sensing of a virus by TLR-3 usually induces pro-inflammatory cytokines, including IL-6, MCP-1, and TNFα. To assess whether SARS-CoV-2 sensing by TLR-3 promotes the release of pro-inflammatory cytokines and amplifies the SASP, senescent human preadipocytes (Figure 3A) and senescent kidney endothelial cells (Supplementary Figure 2) were exposed to the pseudovirus for 96 hrs as well to non-senescent control cells. After exposure, pseudovirus-exposed senescent cells had increased expression of the key SASP factors, IL-1α, IL-1β, IL-8, and chemokine-like CXCL5, compared to non-senescent preadipocytes. This amplification was attenuated by decreasing TLR-3 expression using siRNAs or by treatment with a TLR-3 antagonist (Figure 3B and Supplementary Figure 1). This suggests that TLR-3 activation is sufficient for the pseudovirus to amplify the pro-inflammatory SASP of pre-existing senescent cells.

To determine if senescent cells respond directly to TLR-3 activation, radiation-induced senescent human preadipocytes *vs*. non-exposed senescent controls were exposed to the TLR-3 agonist for 24, 48, or 96 hrs. (Figure 3C). The TLR-3 agonist amplified expression of several cytokines, including IL-1α, IL-1β, IL-6, and IL-8. Expression of these cytokines was higher in exposed senescent cells than cells not exposed to the TLR-3 agonist. Remarkably, the increase in SASP factor expression appeared to be greater after TLR-3 agonist exposure of senescent than non-senescent cells, suggesting that senescent cells have an amplified inflammatory response to RNA-viral PAMPs. Together with the above data showing that a TLR-3 antagonist prevents amplification of the SASP by pseudovirus, these experiments with the TLR-3 agonist indicate that signaling through TLR-3 is both necessary and sufficient for SARS-CoV-2 amplification of the SASP.

### Genuine SARS-CoV-2 amplifies the SASP of senescent preadipocytes cells *via* TLR-3

**Figure 4:**
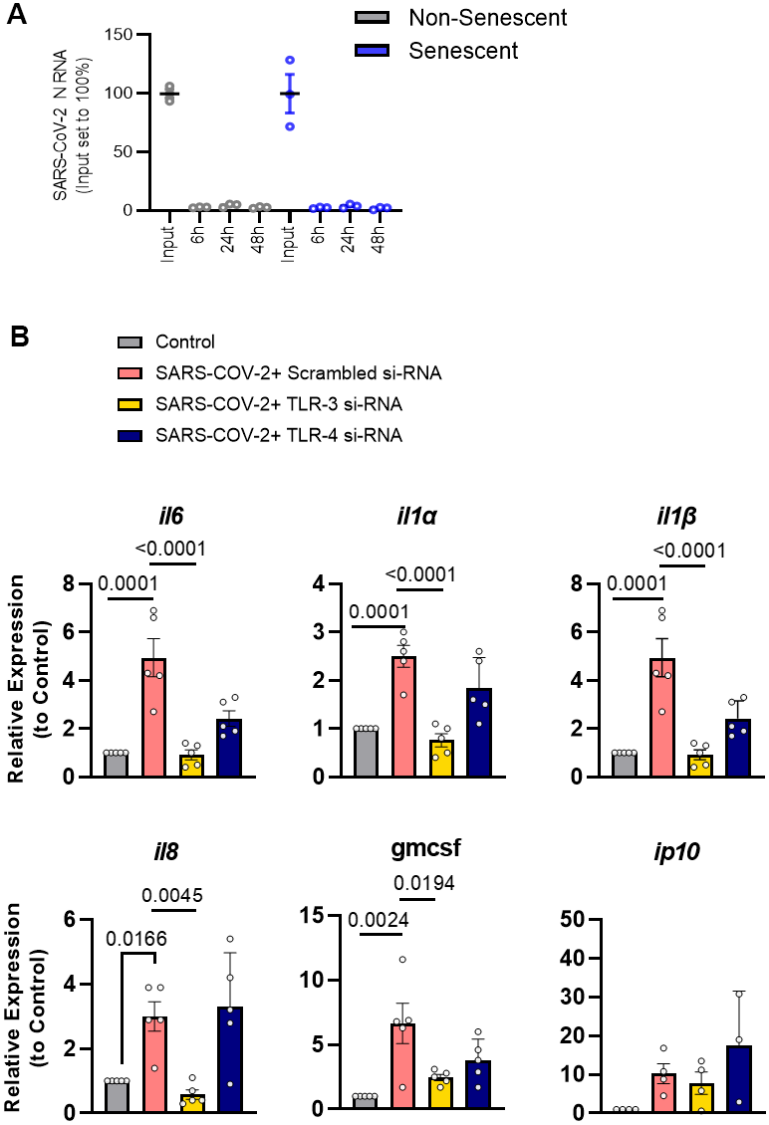
**SARS-CoV-2 amplifies the SASP of senescent preadipocytes without infecting them.** (**A**) Senescent and non-senescent preadipocytes were exposed to SARS-CoV-2 for the indicated times and assayed for infection by qPCR. (**B**) Senescent preadipocytes were treated with SARS-CoV-2 and the SASP was assayed 82 hrs. later by qPCR. Data are expressed as a function of untreated senescent cells; mean +/- SEM, repeated 1-way ANOVA and *post hoc* comparison pairwise Tukey’s HSD. All other significant p values are listed in Supplementary Table 1.

To test if the effects of the pseudovirus reflect the impact of genuine SARS-CoV-2, senescent human preadipocytes were exposed to SARS-CoV-2 for 48 hrs. SARS-CoV-2 did not infect or productively replicate in preadipocytes (Figure 4A). In line with results using pseudovirus or TLR-3 agonists, senescent cells exposed to genuine SARS-CoV-2 virus had an amplified SASP response, characterized by increased IL-1α, IL-1β, IL-6, IL-8, and GMCSF mRNA levels (Figure 4B). Silencing of TLR-3 expression prevented induction of these cytokines, while silencing of TLR-4 had little if any attenuating effect (Figure 4B and Supplementary Figure 1). These results indicate that genuine SARS-CoV-2 external to senescent preadipocytes is sensed in a TLR-3-dependent manner, further exacerbating their pro-inflammatory SASP.

### Lung p16^INK4a+^ senescent cell burden is greater in patients dying from acute SARS-CoV-2 than from other causes

**Figure 5:**
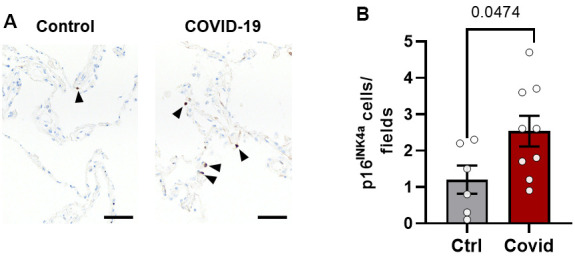
**Lung p16^INK4a+^ senescent cell burden is greater in patients dying from acute SARS-CoV-2 than other causes.** Lung tissue from patients who died from SARS-CoV-2 (n=9) were compared to controls (n=6) who died from other causes without lung disease (see Supplementary Tables 2, 3). (**A**) Paraffin-embedded lung autopsy tissue was sectioned and stained for p16^INK4a^ by immunohistochemistry (black arrowheads). (**B**) Fifteen fields of alveolar tissue were randomly selected and counted. Mean +/- SEM, unpaired 2-tailed Student’s t-test.

To investigate if SARS-CoV-2 infection is associated with induction of senescence, we compared p16^INK4a^ expression in lungs from 10 patients who had died from SARS-CoV-2 (age 76 ±13 years; mean ± SD; 3 females, 7 males) to 6 controls (age 78 ± 19.5 years; 2 females, 4 males) who did not have COPD, asthma, or other pulmonary diseases (Mayo Clinic Tissue Registry; Mayo Clinic IRB #21-001392) as shown in Supplementary Tables 1 and 2. We found increased numbers of p16^INK4a^ positive cells in the lungs of patients who had died from SARS-CoV-2 (Figure 5), consistent with the possibility that SARS-CoV-2 infection can induce cellular senescence.

## DISCUSSION

We previously reported that SARS-CoV-2 surface antigen Spike-1 protein (S1), which signals through ACE2 receptors, can cause amplification of the tissue-destructive, pro-inflammatory SASP of already senescent human cells [3]. Here, we show that SARS-CoV-2 Spike pseudotyped VSV can cause non-senescent cells to become senescent through TLR-3 in kidney endothelial and lung epithelial cells. However, in future studies other cells types also need to be assessed. Additionally, the pseudovirus and genuine SARS-CoV-2 amplified the SASP through TLR-3, with senescent cells becoming more pro-inflammatory than non-senescent cells after viral exposure. Expression of TLR-3 mRNA and protein was higher in senescent than non-senescent cells. Genuine SARS-CoV-2 did not replicate to detectable levels in either senescent preadipocytes or non-senescent preadipocytes, yet the virus amplified the SASP in the former. This is consistent with the increased abundance of TLR-3 receptors on senescent *vs*. non-senescent cells contributing to SASP amplification. Furthermore, TLR-3 antagonists or depletion prevented senescence induction as well as SASP amplification by the pseudovirus and genuine SARS-CoV-2, further indicating a role for TLR-3. A TLR-3 activator amplified the SASP, paralleling effects of viral exposure, suggesting that TLR-3 signaling is both necessary and sufficient for SASP amplification by SARS-CoV-2. This exacerbation of the SASP by SARS-CoV-2 through TLR-3 might further contribute to the SASP induction by S1 that we reported previously in multiple cells to test further the generalizability of the mechanisms we reported [3]. Key findings with the pseudovirus were recapitulated with genuine SARS-CoV-2. Also, more highly p16^INK4a^-expressing cells were present in lungs of patients who had died from SARS-CoV-2 than patients dying from other causes, indicating an increased senescent cell burden in SARS-CoV-2 patients. However, more senescent markers, *e.g*., p21^CIP1^, will need to be examined in patients’ lungs, since p16^INK4a^ expression can also be increased in non-senescent cells, such as activated macrophages [40]. Formation of new senescent cells, coupled with SASP amplification, could contribute to the markedly greater morbidity and mortality from SARS-CoV-2 infection in patients with high pre-existing senescent cell burden than young, previously healthy patients. This includes elderly SARS-CoV-2 patients as well as younger patients with cellular senescence-linked conditions, such as obesity, diabetes, chronic respiratory, cardiovascular, and renal diseases, cancers, or a history of chemotherapeutic or radiation treatment, among others [5, 24, 25, 31, 41].

It has been appreciated for some time that the SASP can be attenuated by “senomorphic” agents, such as rapamycin or metformin [42]. However, it has only recently become apparent that the SASP can be amplified by signals like PAMPs, such as lipopolysaccharide or S1 antigen, as predicted by our SASP “Amplifier/Rheostat Hypothesis” [3]. The finding that SARS-CoV-2 exacerbates the SASP through TLR-3 is consistent with this hypothesis, which may partly explain the increased morbidity and mortality due to hyper-inflammation and tissue destruction in older and chronically-ill individuals compared to previously healthy, younger SARS-CoV-2-infected individuals. Also, consistent with this hypothesis, we recently reported that reducing senescent cell abundance genetically or pharmacologically (with senolytic agents) in old mice infected with a β-coronavirus related to SARS-CoV-2, mouse hepatitis virus (MHV), reduces their higher risk for cytokine storm and mortality than young mice [3]. Potentially, the Amplifier/Rheostat Hypothesis accounts for the increased morbidity and mortality in patients with increased pre-existing senescent cell burden from other types of infections, although further testing is needed.

Interactions between senescent cells, their SASP, and immune cells likely contribute to the impact of senescent cells on SARS-CoV-2 morbidity and mortality [14]. Immune cells can act to increase senescent cell abundance. Activated neutrophils, which accumulate extensively in lungs of severely ill SARS-CoV-2 patients [43], have the capacity to induce non-senescent cells to become senescent [10], potentially adding to the senescent cell burden caused by SARS-CoV-2 through TLR-3 dependent signalling.

Senescent cells are resistant to apoptosis [13] and are mainly removed by the immune system [14]. We previously found that the SASP causes spread of senescence to normal cells, not only locally but also at a distance [20]. These observations led us to propose the “Threshold Theory of Senescent Cell Burden”. This theory, if true, suggests that above a threshold abundance, senescent cells persist and even increase in number because the rate of formation of new senescent cells exceeds senescent cell removal by the immune system. Reaching this threshold would depend on the sum of pre-existing and newly formed senescent cells. To confirm whether such a threshold model would be mathematically tenable given available data and information remains to be evaluated. New senescent cells induced by viral RNA, on top of senescent cell spread from SASP amplification due to virally-induced TLR-3 activation, S1-induced ACE2 stimulation, and increased ROS, could result in surpassing the senescent cell threshold, leading to feed-forward increases in senescent cell burden and resulting morbidity and mortality. Adding to the above, a high burden of senescent cells can impair normal immune system function [14].

A persisting or increasing burden of senescent cells, the SASP, and/or SASP amplification could combine to contribute to both immediate and long-term morbidity due to current or previous SARS-CoV-2 viremia. Consistent with this possibility, transplanting even a small number of senescent cells causes complications resembling those of coronavirus infection in mice, including frailty, weakness, decreased activity, and increased mortality [20]. Hence, increased abundance of senescent cells might contribute to the brain fog/anxiety, physical inactivity/lethargy/muscle weakness/frailty, lung fibrosis/dyspnea, and arthritis/generalized pain symptoms that can persist after acute SARS-CoV-2 infection, the so called post-acute sequelae of COVID-19. Senescent cell burden may even account, in part, for the frailty/accelerated aging-like state that progresses four times faster in older nursing home residents *vs*. uninfected residents for months after SARS-CoV-2 viremia has resolved [44]. Based on these considerations, a clinical study has commenced to test if senescent cell burden is increased in patients with PASC *vs*. age-matched controls (Cellular Senescence and COVID-19 Long-Hauler Syndrome; clinicaltrials.gov identifier NCT04903132).

The effects of SARS-CoV-2 on SASP activation and induction of cellular senescence considered here raise a number of questions that indicate directions for further research, particularly regarding potential clinical interventions to delay, prevent, and treat short- and long-term complications of SARS-CoV-2 viremia. One such class of drugs is senolytics, agents that selectively eliminate senescent cells. Senolytics transiently disable the senescent cell anti-apoptotic pathway (SCAP) network that shields senescent cells from their own SASP and that allows them to survive despite their killing cells around them and causing tissue damage [45–50]. We recently reported that morbidity and mortality in old mice infected with ß-coronavirus, MHV, are attenuated by the senolytics, Fisetin and the combination of Dasatinib and Quercetin [3]. Clinical trials of senolytics for SARS-CoV-2 are already underway, including a SARS-CoV-2 acute hospital trial (COVID-FISETIN: Pilot in SARS-CoV-2 of Fisetin to Alleviate Dysfunction and Inflammation; NCT04476953), a skilled nursing facility trial (COVID-FIS, A Study of Fisetin for Skilled Nursing Facility Residents with COVID-19; NCT04537299), and an outpatient trial (COVFIS-HOME: COVID-19 Pilot Study of Fisetin to Alleviate Dysfunction and Disease Complications; NCT04771611). Senomorphics, such as metformin or agents related to rapamycin, such as sirolimus, offer an alternative way to attenuate the SASP [42]. Trials with senomorphics are also underway (*e.g*., metformin: NCT04510194 and NCT04727424; sirolimus: NCT04341675 and NCT 04948203). Yet another option might be to conduct trials with TLR-3 antagonists, which have been used in pre-clinical studies of SARS-CoV-2 infection [51]. These agents, which have been in clinical trials as immuno-modulators and adjuvants to enhance vaccine effectiveness, have not for the most part advanced past early phase trials [52]. While TLR-3 antagonists may prove to be of use for treating SARS-CoV-2, this might only be during a narrow window early in the course of infection because, based on the findings reported here, these agents would mainly be effective during active viremia before senescent cell abundance has been increased.

## MATERIALS AND METHODS

### Cell culture

Preadipocytes were isolated from abdominal subcutaneous fat biopsies obtained from subjects undergoing gastric bypass surgery. All subjects gave informed consent. The protocol was approved by the Mayo Clinic Institutional Review Board for Human Research. Cells were isolated, cultured, and were made senescent as previously described [53] and corresponding non-senescent were used as controls whenever required. Human primary renal glomerular endothelial cells (Science Cell, Cat #4000, Carlsbad, CA, USA) and human small airway epithelial cells (Cat# CC-2547, Lonza) were purchased and cultured following the manufacturer’s instructions. Human preadipocytes and kidney endothelial cells were made senescent by 20 Gy irradiation and experiments were performed after 30 days and 21 days, respectively.

### Reagents

Cells were treated with following reagents as indicated in the figures: 1) polyinosine-polycytidylic acid (Cat #tlrl-pic-5, Invivogen, San Diego, CA, USA), 2) (R)-2-(3-chloro-6-fluorobenzo[b]thiophene-2-carboxamido)-3-phenylpropanoic acid, Toll-Like Receptor-3/double strand RNA complex inhibitor (Cat# 614310-10MG, Burlington, MA, USA), and 3) pseudovirus (Cat #B2000052, Brainvta, Wuhan, China).

### RNA extraction and rtPCR

For most studies, RNA isolation and rtPCR were performed using Trizol as in [54, 55]. Cells were washed with PBS, then Trizol and chloroform were added to each sample. Samples were centrifuged to separate the aqueous layer. RNA was purified using columns (Qiagen Kit Cat#74104) according to the manufacturer’s instructions. Concentration and purity of samples were assayed using a Nanodrop spectrophotometer. Each cDNA sample was generated by reverse transcription using 1-2000 ng RNA following the manufacturer’s recommended protocol (High-capacity cDNA Reverse Transcription Kit; Cat #4368813, Thermo Fisher Scientific, Waltham, MA, USA). A standard reverse transcription program was used (10 min. at 25° C, 120 min. at 37° C, 5 min. at 85° C, held at 4° C). TBP was used as a control for gene expression analysis. For the experiments related to coronavirus, we used following method for rtPCR. SARS-CoV-2 nucleoprotein (N) RNA levels were assayed in supernatants of infected samples 48 hrs. post-infection. Forward (HKU-NF): 5’-TAA TCA GAC AAG GAA CTG ATT A-3’ and reverse primers (HKU-NR): 5’-CGA AGG TGT GAC TTC CAT G-3’; Probe (HKU-NP): 5’-FAM-GCA AAT TGT GCA ATT TGC GG-3’TAMRA) were from Biomers (Ulm, Germany). IL-6, IL-8, IP-10, CSF2, IL-1α, IL-1β, p16^INK4a^, and p21^CIP1^ primers and probes (TaqMan) were from Thermo Fisher Scientific. RNA levels were determined in cells collected from SARS-CoV-2-infected samples 82 hrs. post-infection. Total RNA was isolated from cells or supernatants using a Viral RNA Mini Kit (#52904, Qiagen, Hilden, Germany) according to the manufacturer’s instructions. rtPCR was performed according to the manufacturer’s instructions using TaqMan Fast Virus 1-Step Master Mix (Cat#4444436, Thermo Fisher Scientific) and a OneStepPlus Real-Time PCR System (96-well format, fast mode). Synthetic SARS-CoV-2-RNA (Cat#Q-87194, Twist Bioscience, South San Francisco, CA, USA) was used as a quantitative standard to determine viral copy numbers. All reactions were run in duplicate. mRNAs were expressed as a function of GAPDH primer/probe sets (Cat#4310884E, Thermo Fisher Scientific). Data were analyzed by the ΔΔCt method.

Primers used are listed below.

**Table:** 

**Gene name**	**Primers**
TLR1	Hs00413978_m1
TLR2	Hs02621280_s1
TLR3	Hs01551079_g1
TLR4	Hs00152939_m1
TLR5	Hs01920773_s1
TLR6	Hs01039989_s1
TLR7	Hs01933259_s1
TLR8	Hs07292888_s1
TLR9	Hs00370913_s1
TLR10	Hs01935337_s1
TBP	Hs00427620_m1
P16	Hs00923894_m1
P21	Hs00355782_m1
IL-1α	Hs00174092_m1
IL-1β	Hs01555410_m1
Il-6	Hs00174131_m1
IL-8	Hs00174103_m1
IP-10	Hs00171042_m1
CSF2	Hs00929873_m1

### siRNA knockdown

Cells were transfected with the indicated siRNAs using RNAi max reagent (Cat#13778075; Thermo Fisher Scientific) as in [55]. Briefly, cells were transfected in antibiotic-free media in 6 well plates. 9μl RNAi max/well were mixed in 150μl OPTI-mem media and 6μl 10μM siRNA in 150μl of OPTI-MEM medium in a separate Eppendorf tube. The two tubes were mixed and incubated for 5 mins. 250μl of the mixture were added to wells. The following siRNAs were purchased from Thermo Fisher Scientific): TLR-3 siRNA (Assay ID: 107054) and TLR-4 siRNA (Assay ID: s-14195).

### Western blots

Cells or tissues were homogenized in RIPA buffer (Cat #89900, Thermo Fisher Scientific) with protease inhibitors (Cat# 78430, Thermo Fisher Scientific). Proteins were loaded on SDS-PAGE gels and transferred to immuno-blot PVDF membranes (Biorad, Hercules; CA, USA). ECL Western Blotting Substrate (Pierce; cat #32106, Rockford IL, USA) was used to develop signals. TLR-3 (catalog #6961) and α-tubulin (catalog #2144) antibodies were purchased from Cell Signaling. Data were quantified using the optical densities of the specified proteins as a function of α-tubulin.

### SARS-CoV-2 stock production

BetaCoV/France/IDF0372/2020 was propagated on Vero E6 infected at a multiplicity of infection (MOI) of 0.003 in serum-free medium containing 1μg/ml trypsin, as previously described [56]. Briefly, the cells were inoculated for 2 hrs. at 37° C before the inoculum was removed. The supernatant was harvested 48 hrs. post-infection upon visible cytopathic effect. To remove debris, the supernatants were centrifuged for 5 min. at 1,000 x g, then aliquoted and stored at −80° C.

### Plaque-forming unit assays

Plaque-forming unit (PFU) assays were performed as previously described [57]. Briefly, SARS-CoV-2 stocks were serially diluted and confluent monolayers of Vero E6 cells infected. After incubation for 2 hrs. at 37° C with shaking every 20 min., the cells were overlaid with 1.5 ml 0.8% Avicel RC-581 (FMC/DuPont; Wilmington, DE, USA) in medium and incubated for 3 days. Cells were fixed with 4% PFA at room temperature for 45 min. After cells had been washed with PBS once, they were incubated in 0.5ml staining solution (0.5 % crystal violet and 0.1 % triton in water) at room temperature. After 20 min., the staining solution was rinsed off with water, virus-induced plaque formation quantified, and PFU/ml calculated.

### Multiplex ELISA

CM was filtered and cytokine and chemokine protein levels in CM were measured using Luminex xMAP technology as in [3]. Multiplexing analysis was performed using a Luminex 100 system (Luminex, Austin, TX, USA) by Eve Technologies Corp. (Calgary, Alberta, Canada). Data are represented as pg/ml for each SASP factor as a function of cellular density.

### Human lung studies

Lungs from 9 patients who had died with SARS-CoV-2 (age 74 ±12 years; mean ± SD; 3 females, 6 males) were compared to 6 controls (age 78 ± 19 years; 2 females, 4 males) who did not have COPD, asthma, or other pulmonary diseases (Mayo Clinic Tissue Registry; Mayo Clinic IRB #21-001392). Paraffin-embedded lung tissue was sectioned into 4μm sections, stained immunohistochemically for p16^INK4a+^ cells (Clone E6H4, #705-4793, Roche Tissue Diagnostics, Indianapolis, IN, USA) using a VENTANA Discovery ULTRA instrument (Ventana Medical Systems; Oro Valley, AZ USA), counterstained with hematoxylin, and scanned using a 40x objective Motic Slide Scanner (Motic Company; Xiamen, China). Images were virtually sliced into 300 x 400 μm numbered fields that were selected using a Microsoft Excel random number generator. Fifteen fields of p16^INK4a+^ lung cells were so counted/subject. Results were analyzed by an unpaired 2-tailed t-test.

### Statistical analysis

All figures were plotted using Prism 9.0 (GraphPad). P value ≤ 0.05 (two-sided) was considered statistically significant. Student’s t-test was used to compare the equality of means from two independent samples, and Welch’s correction was performed when two samples were determined to have significantly unequal variances (Levene’s test). One-way ANOVA was used to compare means from three or more samples, and two-way ANOVA was used when there were two predictors. Tukey’s Honestly Significant Difference (HSD) was used for *post hoc* pairwise comparisons where 4 means were being compared, while Fisher’s Least Significant Difference (LSD) procedure was used when comparing 3 means. Paired t-tests and repeated measures ANOVA were used to account for nesting, *i.e*. correlated data across cells (preadipocytes) from the same subject. All p values (<0.05) are indicated in the figures and Supplementary Table 1.

## Supplementary Material

Supplementary Figures

Supplementary Tables
