# Supplementary material for: SARS-CoV-2 causes senescence in human cells and exacerbates the senescence-associated secretory phenotype through TLR-3
Source: Aging (Albany NY). 2021 Sep 16;13(18):21838–54. doi: 10.18632/aging.203560 (PMC8507266; doi:10.18632/aging.203560)
Supplement: Supplementary Figures [file aging-13-203560-s001.pdf]

SUPPLEMENTARY FIGURES

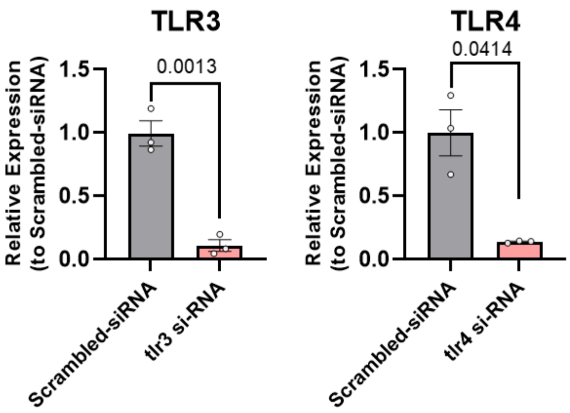

Supplementary Figure 1. siRNA mediated knockdown of TLR-3 and TLR-4 in senescent preadipocytes. Knockdown by siRNA was confirmed by rtPCR after 2 days in senescent preadipocytes (n=3). Data are expressed as a function of scrambled siRNA-treated senescent cells; mean +/- SEM, paired 2-tailed Student's t-tests.

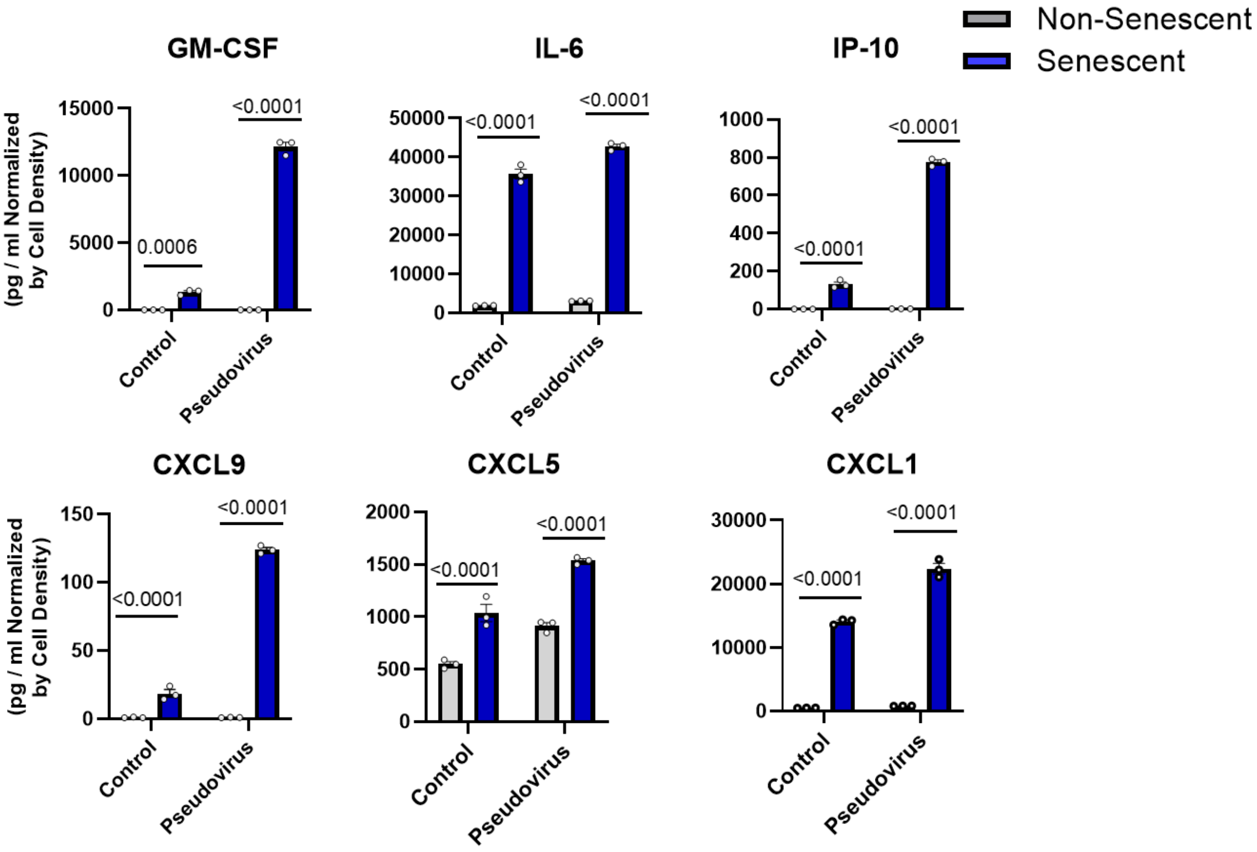

Supplementary Figure 2. Pseudovirus amplifies the SASP in senescent kidney endothelial cells. SASP factors were assayed in senescent and non-senescent kidney endothelial cells treated with pseudovirus for 96 hrs. Data are shown as a function of cell number; mean +/- SEM, 2-way ANOVA and *post hoc* comparison Fisher's LSD.
