# Supplementary material for: SARS-CoV-2 causes senescence in human cells and exacerbates the senescence-associated secretory phenotype through TLR-3
Source: Aging (Albany NY). 2021 Sep 16;13(18):21838–54. doi: 10.18632/aging.203560 (PMC8507266; doi:10.18632/aging.203560)
Supplement: Supplementary Tables [file aging-13-203560-s002.pdf]

SUPPLEMENTARY TABLES

Supplementary Table 1. Significant p values in addition to those shown in Figures 3, 4.

Figures	Comparisons	p values
3 B	IL-1β, TLR-3 antagonist *vs.* TLR-3 siRNA	0.0114
	IP-10, TLR-3 antagonist *vs.* TLR-3 siRNA	0.0175
3 C	Il-1α senescent, 24 *vs.* 96 hrs.	0.0466
4 B	Il-1α, TLR-4 siRNA *vs.* control	0.0132
	IL-6, TLR-3 siRNA *vs.* control	0.0450
	IL-6, TLR-4 siRNA *vs.* control	0.0193
	IL-8, TLR-4 siRNA *vs.* control	0.0065
	IP-10, TLR-4 siRNA *vs.* control	0.0288

Supplementary Table 2. Information about the control lung biopsy patients in Figure 5.

Gender	Cause of death	Age
F	Car accident	92
F	Car accident	100
M	Myocardial infarction (from atherosclerotic cardiovascular disease)	52
M	Drowning. Lungs without significant diagnostic abnormalities. No *rigor mortis* (death less than 24 hrs.)	72
M	Cerebrovascular accident	92
M	Dementia/malnutrition	59

Supplementary Table 3. Information about the COVID lung biopsy patients in Figure 5.

Gender	Cause of death/specified COVID complication	Age
M	Respiratory failure due to COVID	71
M	Developed severe hypoxemia. Bronchoscopy did not reveal clear reversible cause	73
M	SARS-CoV-2 bronchopneumonia, lethargic, generalized muscle weakness, hypotension, and continued hallucinations	93
F	Aspiration pneumonia in the setting of a recent SARS-CoV-2 infection. The deceased began antibiotic therapy, however patient experienced significant decline in mentation and renal function	89
M	Acute respiratory distress syndrome (ARDS). Family elected to have compassionate ventilator withdrawal	72
F	COVID-19 associated pneumonia in the setting of multiple chronic medical conditions and missed hemodialysis	71
F	COVID-19 infection requiring intubation, multiple strokes with residual left hemiparesis, diabetes mellitus type 2, coronary artery disease, essential hypertension, acute respiratory failure, and cognitive decline	70
M	CT chest showed findings consistent with COVID infection with profound hypoxemia. Palliative care was consulted and patient transitioned to DNR	76
M	Multiple GI bleeds. Pneumothorax infections. Massive transfusion and death	51
